# The phenotype of monocyte subtypes and expression of chemokine receptors in the Chronic Venous Insufficiency

**DOI:** 10.1016/j.clinsp.2025.100595

**Published:** 2025-02-27

**Authors:** Danielle Borges Germano, Larissa Namie Chiba, Ana Paula Augusto da Cruz Ballerini, Jônatas Bussador do Amaral, Marina Tiemi Shio, Jhefferson Miranda Alves, Daniela Alves de Abreu, Luiz Henrique da Silva Nali, Francisco Antonio Helfenstein Fonseca, Maria Cristina Izar, André Luis Lacerda Bachi, Carolina Nunes França

**Affiliations:** a Post-Graduation Program in Health Sciences, Universidade Santo Amaro, São Paulo, SP, Brazil; b Urology Program, Faculdade de Medicine, Universidade de São Paulo, São Paulo, SP, Brazil; c ENT Research Lab., Department of Otorhinolaryngology Head and Neck Surgery, Universidade Federal de São Paulo, São Paulo, SP, Brazil; d Universidade Federal de São Paulo, São Paulo, SP, Brazil

**Keywords:** Chronic venous insufficiency, Monocyte subtypes, Chemokine receptors

## Abstract

**Objective:**

The aim of this study was to evaluate monocyte subtypes and the chemokine receptors, comparing individuals with and without Chronic Venous Insufficiency (CVI).

**Method:**

The authors included patients undergoing surgery for varicose veins of the lower limbs (Classification of Venous Disorders – CEAP: C1‒C3).

**Results:**

There were increased levels of classical and non-classical monocytes in the case group and a reduction in intermediate monocytes. Besides, there was higher expression of CCR5 in the non-classical monocytes and lower expression of CX3CR1 in the classical monocytes in the case group.

**Conclusions:**

The inflammatory process present in patients with CVI can modulate the monocyte phenotype and the expression of the chemokine receptors at the protein level.

## Highlights

• Receptors have chemotactic property cells and are characterized as inflammatory markers.
• Monocyte subtypes can play an important role in the Chronic Venous Disease scenario.
• Receptors that act by recruiting monocytes include CCR2, CCR5, and CX3CR1.
• Chronic Venous Insufficiency changes the monocyte phenotype.
• The chemokine receptors might be changed by the Chronic Venous Insufficiency.

## Introduction

Chronic Venous Insufficiency (CVI) of the lower extremities is a disease of the circulatory system that has several clinical situations from asymptomatic but aesthetic problems to severe symptoms that may cause signs and symptoms of pain, the sensation of heaviness in the lower limbs and cramps. Around 150,000 new patients in the world are diagnosed with chronic venous insufficiency per year, and nearly $500 million is used in the care of these individuals. Disability-related to CVI has a significant socioeconomic impact, reduced quality of life, and loss of work productivity, especially due to the irreversible sequels caused or promoted by the condition, including venous leg ulcers.^1^ In addition, quantification of the incidence and prevalence of CVI is difficult because there is a wide range of symptoms, and protocols used for diagnosis, and the words used to express the feeling of discomfort by patients with venous disorders vary between countries.^2^

CVI usually refers to the venous reflux or dysfunction of the superficial venous system to venous hypertension, caused by valve incompetence and/or venous outflow obstruction.^2^ CVI can be developed when venous hypertension of the lower extremities occurs because of venous valve incompetence, venous obstruction, or a combination of both. Undiminished venous hypertension can result in dermal changes with hyperpigmentation, fibrosis of subcutaneous tissue, termed “lipodermatosclerosis” and eventual ulceration.^3^ The inflammatory process is characterized as the immune response that begins in the tissue in a situation of injury and involves the reaction of blood vessels, causing the accumulation of fluids and blood cells of the immune system, mobilizing them.^4^

Some receptors have the chemotactic property of peripheral blood mononuclear cells and are characterized as inflammatory markers, where in many chronic diseases it is possible to identify tissue infiltrates of lymphocytes and, especially, monocytes. Receptors that act by recruiting monocytes to sites of trauma, injury, inflammation, and ischemia include CCR2 (C-C chemokine Receptor type 2), CCR5 (C-C chemokine Receptor type 5), and CX3CR1 (CX3C chemokine Receptor 1), among others.^4^

Monocytes are characterized by the expression of the Lipopolysaccharide receptor (LPS) CD14 and CD16, FcγIII receptor, fundamental components of innate immunity and also play an important role in inflammation. Based on the expression of CD14 and CD16 Lipopolysaccharide receptors (LPS), monocytes are classified into 3 types: classical monocytes (CD14++CD16-), intermediate monocytes (CD14++CD16+), and non-classical monocytes (CD14+CD16++).^5^

In this sense, the aim of this study was to evaluate monocyte subtypes (classical, intermediate and nonclassical) as well as the chemokine receptors (CCR2, CCR5, and CX3CR1), comparing individuals with and without CVI.

## Materials and methods

### Study design and patients

This is a case-control study that included patients undergoing surgery for varicose veins of the lower limbs in the Santa Casa de Misericórdia de Santo Amaro, São Paulo – Brazil (Classification of Venous Disorders – CEAP: C1-C3), and a control group of patients who did not have CVI, after signing the free and informed consent form of volunteer patients. The exclusion criterion was applied to those who did not exhibit appropriate clinical conditions to respond to research instruments or dropped out after starting the search.

The study followed the STROBE guidelines.

The local Ethics Committee approved the study (protocol number: 3.041.271.

### Phenotypic characterization of monocyte subtypes

The samples were collected in tubes with EDTA to prevent clotting. Peripheral blood mononuclear cells (PBMC) were obtained by concentration gradient (Ficoll Paque Plus, GE Healthcare Bio-Sciences AB, Uppsala, Sweden).

PBMC were centrifuged and immunostained with the following antibodies: CD14 conjugated with Allophycocyanin ‒ APC (BD Biosciences, Franklin Lakes, NJ, USA) and CD16 conjugated with Fluorescein Isothiocyanate ‒ FITC (BD Biosciences, Franklin Lakes, NJ, USA). As controls, cells labeled with the isotypes IgG1 APC (BD, Biosciences, Franklin Lakes, USA) and IgG1 FITC (BD Biosciences, Franklin Lakes, NJ, USA) were analyzed. Immediately after this process, the reading was performed in a flow cytometer (FACSCalibur – BD Biosciences, San Jose, USA) with analysis performed by the Cell Quest Pro software. Monocyte subtypes were expressed as a percentage (%), with approximately 50,000 events being acquired.^5^

### RNA isolation and reverse transcription

The RNA isolation and Reverse Transcription were developed as previously published.^4^ Briefly, the PureLink RNA Mini Kit (Thermofisher) was used for RNA isolation, following the manufacturer's recommendations.

The RNA present in each sample was obtained with the Nanodrop 2000 device (Applied Biosystems).

The Superscript II Reverse Transcriptase kit (Thermofisher) was used to obtain the cDNA (reverse transcription).

The GAPDH gene was used as the control gene.

### Expression of the genes for CCR2, CCR5 and CX3CR1

For evaluation of the expression of the genes for CCR2, CCR5 and CX3CR1, the Real-time PCR StepOnePlus system (Thermo Fisher Scientific) was used.

Primers for CCR2 were forward 5′-ATGCTGTCCACATCTCGTTCTCG-3′, reverse 5′-TTATAAACCAGCCGAGACTTCCTGC-3′.^4^

Primers for CX3CR1 were: forward 5′-CCCTGAATCAGTGACAGAAAACT-3′; reverse 5′-ACGGAGTAGAATATGGACAGGAA-3′.^4^

The primers for CCR5 were: forward 5′-CACCTGCAGCTCTCATTTTCC-3′; reverse 5′-TTGTAGGGAGGCCCAGAAGAG-3′.^4^

### Statistical analysis

SPSS version 26.0 was used for data analysis. Parametric and non-parametric tests were used, according to the nature of the variables. The significance level was set at p < 0.05. To compare the central tendencies of two samples independent of different sizes, the non-parametric Mann-Whitney test was used.

## Results

The study included a case group (n = 47), 98% were female; the mean age (standard deviation – SD) was 47 (10), and the median (interquartile range) was 48 (39‒53). In the group control (n = 61), 77% were women, there was a mean age (SD) of 50 (14), and the median (interquartile range) was 48 (38‒59). There was a higher percentage of women in the case group (p = 0.001, Chi-Square test), with no significant differences between age groups (p = 0.2) (data not shown).

**Fig. 1:**
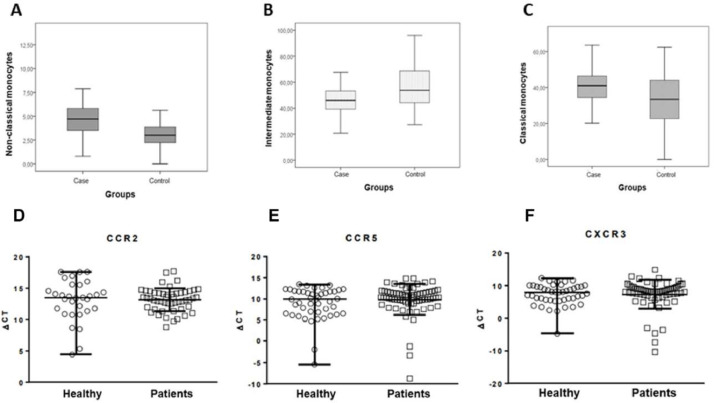
Classical (A), intermediate (B), and non-classical (C) monocyte, comparing case and control groups. Mann-Whitney test, p < 0.0001, p = 0.019, and p = 0.002, respectively. CCR2 (D), CCR5 (E) and CX3CR1 (F) monocyte chemokine receptor expression in case and control groups. Mann-Whitney test, p = 0.73, p = 0.17, and p = 0.38, respectively.

Fig. 1 represents the expression levels of receptors for monocyte chemokines (CCR2, CCR5 and CX3CR1, respectively) in case and control groups, as well as the levels of monocyte subtypes (classical, intermediate, and non-classical monocytes) in both groups. There were no significant differences for the receptors (p = 0.73; p = 0.17 and p = 0.38, respectively. Mann Whitney test). After comparisons between groups, there were increased levels of classical and non-classical monocytes in the case group and a reduction in intermediate monocytes (p < 0.0001, p = 0.019, and p = 0.002, respectively. Mann-Whitney test).

Table 1 shows the level of expression of the chemokine receptors in the monocyte subtypes. There was higher expression of CCR5 in the non-classical monocytes in the case group and lower expression of CX3CR1 in the classical monocytes in the case group (p < 0.0001 and p = 0.009, respectively. Mann-Whitney Test), without differences for CCR2. There were also no differences in the control group.

**Table 1:** Level of expression (a.u.) of the receptors CCR2, CCR5, and CX3CR1 in the monocyte subtypes, comparisons between the case and the control groups.

Monocytes	Case	Control	p between groups^a^	p between groups^b^	
				Case	Control
***CCR2***					
Classical	151.18 (124.13‒173.27)	151.47 (121.57‒172.96)	0.499		
Intermediate	310.52 (272.07‒360.47)	308.97 (262.93‒360.65)	0.276	<0.0001	<0.0001
Non-classical	127.98 (108.37‒149.12)	129.13 (111.40‒148.74)	0.925		
***CCR5***					
Classical	89.51 (58.87‒127.82)	89.38 (53.53‒131.91)	0.903		
Intermediate	357.26 (250.23‒444.62)	340.28 (246.10‒407.38)	0.090	<0.0001	<0.0001
Non-classical	215.02 (139.75‒281.47)	190.59 (119.68‒252.84)	<0.0001		
***CX3CR1***					
Classical	75.23 (53.00‒100.13)	84.97 (61.69‒11.97)	0.009		
Intermediate	247.72 (133.31‒352.64)	231.76 (149.13‒346.77)	0.930	<0.0001	<0.0001
Non-classical	53.99 (36.22‒84.76)	52.72 (33.45‒85.53)	0.364		

Data represent medians (interquartile interval).

a Mann Whitney test, between group comparisons (Case × Control).

b Kruskal-Wallis test, comparisons between groups (Classical × Intermediate × Non-classical).

## Discussion

The main objectives of the present study were to compare not only the percentage of monocyte subtypes but also the expression of the chemokine receptors CCR2, CCR5, and CX3CR1, both in the mRNA as protein level, in CVI patients (case group) and a control group (without CVI).

Regarding the literature, among some mechanisms involved in hypertension and vascular changes, it highlights the leukocyte-endothelium interaction, particularly by monocytes infiltrating the valve and venous wall, which promote their destruction and remodeling. In fact, the monocytes are pivotal in generating the inflammation that can lead to acquired valve dysfunction, and chronic venous disease patients show infiltration of valve leaflets and the venous wall by this immune cell.^6^^,^^7^

Here, the authors found an increase in classical and non-classical monocytes and a decrease in the intermediate subtype in the case group when compared to the control group in the current study.

Höpfner et al.^8^ showed a greater number of classical monocytes in patients hospitalized with Coronary Artery Disease (CAD), pointing to the pathophysiological role of blood monocytes in the inflammatory scenario. According to the study by Berg et al.,^9^ elevated levels of classical monocytes were found in a group with cardiovascular disease, in comparison with a control group, which is in accordance with the present study.

Despite controversies regarding the role of intermediate monocytes, the study by Lo et al.^10^ showed that this subtype may indicate an increased risk in asymptomatic patients with plaque or severe coronary stenosis. Intermediate monocytes have been considered cells in a transitory stage of differentiation, which can give rise to other subtypes, depending on the microenvironment to which patients are subjected. In this study, however, the authors found lower levels of intermediate monocytes in the case group, which need to be evaluated in large studies involving individuals with CVI.

No significant differences were found in the expression of CCR2, CCR5, and CX3CR1 at the mRNA level when comparing the case and control groups in the present study. Although the recent study performed by Vodovotz et al.^11^ can, in part, corroborate the findings, since the authors did not observe significant differences in some chemoattractant molecules comparing CVI patients and the control group, as far as the authors know, these findings represent an intriguing novelty in the scenario of CVI.

Some studies analyzed the expression of these receptors but associated with Acute Myocardial Infarction and/or the development of atherosclerotic plaques, where there was a greater relationship between increased expression and the development of cardiovascular diseases, with results dependent on the association with factors such as plasma concentration of low-density lipoprotein cholesterol (LDL-C).^4^

In a study carried out by Verweij et al. in 2017,^12^ the relationship between the expression of CCR2 by monocytes and the concentration of Low-Density Lipoprotein Cholesterol (LDL-C) in a population at risk of CVD was shown. LDL-C is capable of stimulating the overexpression of monocyte ligand CCL2, thus providing a link between upregulation for CCR2 receptors and hypercholesterolemia.

Corroborating such data, another analysis carried out by Grune et al. in 2017^13^ determined that LDL-C facilitates monocyte chemokine MCP-1-dependent chemotaxis through the induction of CCR2, mediating monocyte function, showing the fundamental role of CCR2 in targeting monocytes to sites of inflammation.

In a murine model of venous thrombosis, the CCR2 expression in monocytes could indicate a pro-inflammatory phenotype is present and that the increase in this receptor and the consequent cell recruitment to venous thrombi improved thrombus clearance.^14^

Similar to CCR2, studies suggest that the chemokine receptor CX3CR1 also appears to be influenced by high-fat diets. Furthermore, a significant reduction in circulating levels of the CX3CR1 ligand, fractalkine, has been shown in congestive heart failure in patients treated with Reconstituted HDL (rHDL), a lipoprotein known to have anti-inflammatory properties.^15^

On the other hand, when the authors compared the expression levels of the receptors CCR2, CCR5, and CX3CR1 at the protein level in the monocyte subtypes in the case and control groups, we observed higher expression of the CCR5 receptor in non-classical monocytes and lower expression of the CX3CR1 receptor in classical monocytes compared to other subtypes in the case group. The findings concerning the modulation of CCR5 and CX3CR1 are in line with a recent study published by the group,^5^ in which patients after Acute Myocardial Infarction showed a persistence of the inflammatory phenotype, known as trained immunity, even after the most effective lipid-lowering and antiplatelet treatments.

The results suggest that the inflammatory process present in patients with Chronic Venous Insufficiency (CVI) can modulate the monocyte phenotype, as well as the expression of the chemokine receptors at the protein level, although without influence by the expression of the monocyte chemokine receptors at mRNA level, after comparison of individuals with and without CVI. However, additional case-control studies involving a larger number of participants are needed, as well as clinical trials evaluating CVI treatment effects associated with the monocyte subtypes and the expression of chemokine receptors.

## Funding

None.

## CRediT authorship contribution statement

**Danielle Borges Germano:** Methodology, Writing – review & editing, Project administration. **Larissa Namie Chiba:** Methodology, Writing – review & editing. **Ana Paula Augusto da Cruz Ballerini:** Conceptualization. **Jônatas Bussador do Amaral:** Validation, Writing – review & editing. **Marina Tiemi Shio:** Validation, Writing – review & editing. **Jhefferson Miranda Alves:** Methodology. **Daniela Alves de Abreu:** Methodology. **Luiz Henrique da Silva Nali:** Writing – review & editing. **Francisco Antonio Helfenstein Fonseca:** Validation. **Maria Cristina Izar:** Validation. **André Luis Lacerda Bachi:** Validation, Supervision. **Carolina Nunes França:** Conceptualization, Data curation, Supervision, Project administration, Funding acquisition.

## Declaration of competing interest

The authors declare that they have no known competing financial interests or personal relationships that could have appeared to influence the work reported in this paper.
